# Editorial: Innovative approaches for precise identification and control of ticks and tick-borne pathogens

**DOI:** 10.3389/fvets.2025.1591609

**Published:** 2025-03-31

**Authors:** Mourad Ben Said, Rosa Estela Quiroz-Castañeda

**Affiliations:** ^1^Higher Institute of Biotechnology of Sidi Thabet, University of Manouba, Manouba, Tunisia; ^2^Laboratory of Microbiology at the National School of Veterinary Medicine of Sidi Thabet, University of Manouba, Manouba, Tunisia; ^3^Centro Nacional de Investigación Disciplinaria en Salud Animal e Inocuidad, INIFAP, Jiutepec, Morelos, Mexico

**Keywords:** ticks, pathogen detection, molecular techniques, genetic characterization, management strategies, innovative approaches

**Figure 1:**
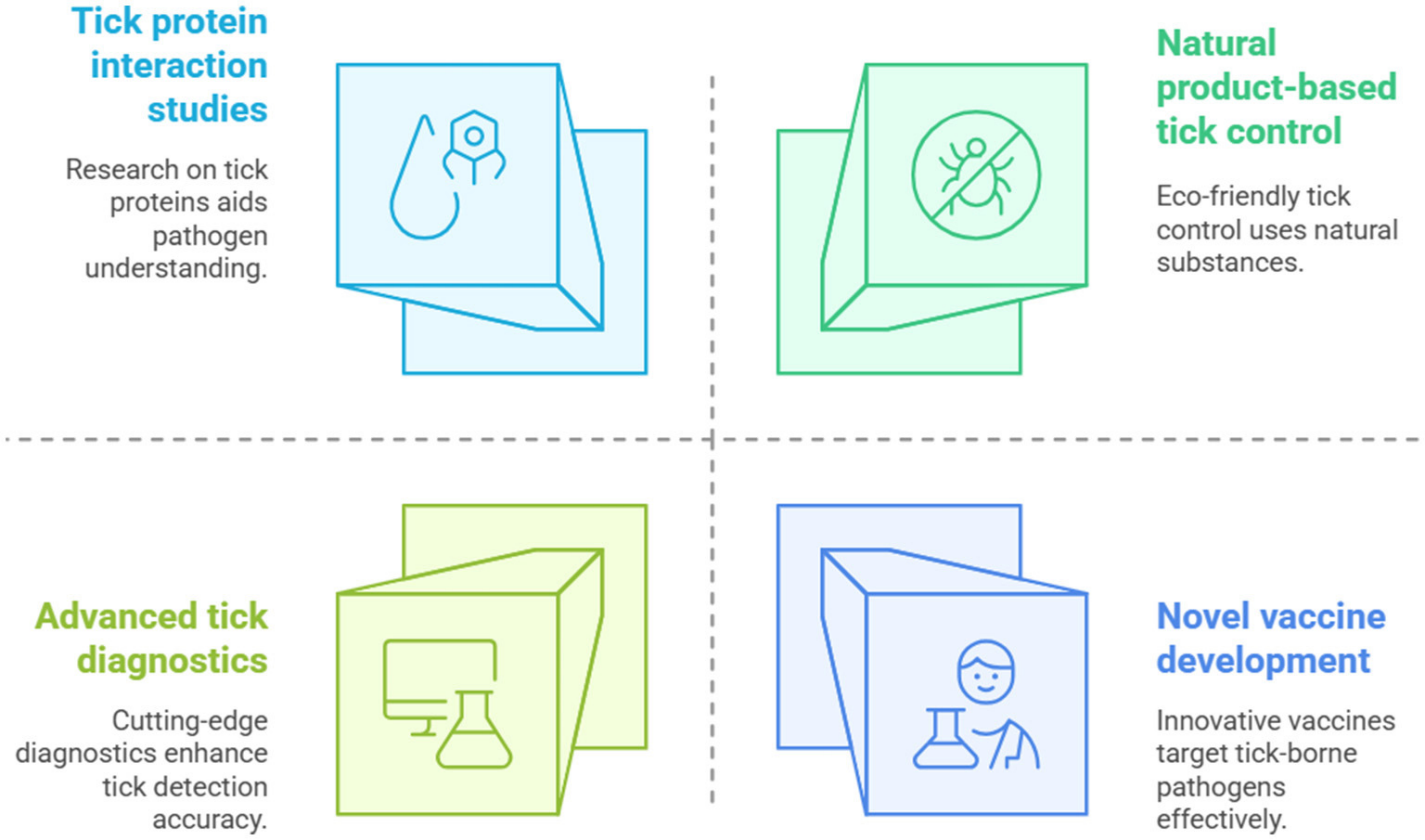
Comprehensive strategies for managing ticks and tick-borne diseases presented in this Research Topic.

This Research Topic presents a collection of original and review articles that explore the diverse aspects of tick and tick-borne disease management. It addresses (i) innovative tick control methods using natural products, (ii) advancements in tick diagnostics and pathogen detection, (iii) novel vaccine strategies for combating vector-borne diseases, and (iv) in-depth research on tick proteins and their roles in pathogen interactions. Together, these contributions enhance our understanding of ticks and their impact on animal and public health, paving the way for effective management strategies (Figure 1).

Innovative and sustainable solutions are crucial in the ongoing fight against tick infestations and the diseases they transmit. The study by Abd-Elrahman et al. explore the effectiveness of natural extracts, specifically Chrysanthemum extract and neem oil emulsion, for controlling tick populations and cattle-associated infections. Their findings indicate a significant relationship between tick infestations and the presence of pathogens such as *Theileria annulata* and *Babesia bigemina*, highlighting the critical role ticks play in disease transmission. The research also demonstrates the potential of these natural products as effective alternatives to conventional acaricides. Complementing this work, Phaahla et al. investigate the traditional knowledge and practices used by local communities in Sekhukhune District, South Africa, for managing cattle ticks. Their study emphasizes the value of integrating traditional, plant-based methods into livestock health care, especially in resource-limited settings. By engaging with community members, they identified numerous plant species with potential acaricidal properties, advocating for eco-friendly farming practices and preserving indigenous knowledge. These insights support the conservation of traditional practices and suggest ways to incorporate ethnoveterinary methods into modern tick control strategies.

In diagnostics and pathogen detection, Kamau et al. focus on identifying *Coxiella burnetii* DNA in ticks, emphasizing the zoonotic risks of Q fever linked to infected livestock and wildlife. Their study evaluates three diagnostic methods: conventional PCR (cPCR), Biomeme's *C. burnetii* qPCR Go-strips, and a novel PCR high-resolution melt (PCR-HRM) assay. Analyzing ticks collected from wildlife and cattle in northern Kenya, the researchers used Bayesian latent class analysis (BLCA) to assess the sensitivity and specificity of these tests without a gold standard. The results showed that the PCR-HRM assay had the highest sensitivity at 86%, followed by the Biomeme test at 57% and cPCR at only 24%, while all methods maintained high specificity (94% to 98%). Notably, about 16% of the ticks tested positive for *C. burnetii* DNA, indicating the endemic presence of this pathogen in the area. This suggests that PCR-HRM could serve as a reliable tool for *C. burnetii* surveillance in tick populations, offering valuable insights for future epidemiological research and public health initiatives. Additionally, the study by Sri-in et al. examine tick diversity and their role as vectors for various pathogens, including *Anaplasma, Babesia*, and *Theileria*. Conducted at Khao Kheow Open Zoo in Thailand, this research involved the collection of over 10,000 ticks over a year, revealing a high pathogen infection rate of 66.37%, with *Anaplasma* spp. being the most prevalent at 55.23%. This work highlights the importance of understanding tick diversity and pathogen prevalence as indicators of wildlife health and ecosystem dynamics.

Regarding immunization against vector-borne diseases, Alzan et al. offer a comprehensive overview of current vaccines, experimental trials, and innovative strategies to control veterinary-relevant blood parasites. These parasites, transmitted by ticks and other blood-sucking arthropods, pose substantial threats to human and animal health, exacerbated by environmental changes. The infections they cause can lead to a range of clinical symptoms, including fever, anemia, jaundice, and neurological disorders, with varying mortality rates. The review emphasizes the need for effective control measures primarily involving vector control, drug treatments, and vaccination strategies. However, many existing approaches face limitations, such as environmental concerns linked to the use of parasiticides and the practicality of vaccine deployment in non-endemic areas. The authors highlight recent advances in vaccine development, particularly those utilizing recombinant antigens, vectored vaccines, and live attenuated or genetically modified parasites. Despite ongoing research, the challenge of creating effective subunit vaccines against blood-stage parasites persists. The authors advocate for leveraging insights from previous vaccine development efforts and employing emerging technologies to refine immune mechanisms of protection, identify appropriate adjuvants, and discover protective antigens, thus enhancing the arsenal against these significant veterinary pathogens. In a complementary study, Quiroz-Castañeda et al. explore an alternative vaccine target for bovine anaplasmosis, focusing on enolase, a moonlighting protein. This research shifts away from traditional vaccine targets, such as Major Surface Proteins and Outer Membrane Proteins, toward multifunctional proteins that can perform various biological roles. The study identifies three enolase proteins in Mexican strains of *Anaplasma marginale* and employs bioinformatics to predict their catalytic domains and binding capabilities. Importantly, the molecular docking analysis indicates that one of these enolases, AmEno01, may interact with erythrocyte proteins, which is crucial for the pathogen's adhesion and invasion.

Focusing on tick biology and diagnostics, Li et al. examine the proteins in the hemolymph of *Haemaphysalis flava* ticks, providing important insights into the biological mechanisms that affect these ectoparasites. Their study emphasizes the role of hemolymph as a circulating fluid that transports nutrients, immune factors, and waste, with proteins being its main soluble components. Using blue native polyacrylamide gel electrophoresis (BN-PAGE) and sodium dodecyl sulfate PAGE (SDS-PAGE), the researchers identified critical proteins, particularly from the vitellogenin (Vg) family and α-macroglobulin. These protein identifications are vital, as they may play significant roles in tick physiology and immunity, presenting potential targets for tick management strategies. In another study, Tila et al. report the first detection of *Hepatozoon ayorgbor* in *Rhipicephalus haemaphysaloides* and *Hepatozoon colubri* in *Haemaphysalis sulcata* and *Hyalomma anatolicum*. This research highlights the risks of pathogen spillover from wildlife to domestic animals, stressing the importance of vigilance in monitoring tick-borne diseases. Their study involved extensive tick collection and molecular analysis, revealing significant infection rates and identifying environmental risk factors for spillover events. Additionally, Zamiti et al. contribute to improving tick diagnostics by developing PCR assays that utilize minimal *Bm*86 cDNA fragments to identify *Rhipicephalus* and *Hyalomma* tick species. This research addresses the limitations of traditional morphological identification methods, providing a sensitive and specific molecular tool for tick species delineation. By concentrating on shorter, targeted sequences, the study enhances our ability to effectively classify ticks, which is crucial for managing the spread of tick-borne diseases.

As Editors of this Research Topic, we would like to extend our gratitude to all authors for their valuable contributions of original research and insightful reviews. We believe that readers will discover this Research Topic to be an essential reference on recent advancements in the field, covering (i) cutting-edge tick control strategies utilizing natural compounds, (ii) progress in tick diagnostics and pathogen identification, (iii) innovative vaccine approaches for addressing vector-borne diseases, and (iv) detailed investigations into tick proteins and their functions in pathogen interactions.

